# Hybrid Composites Based on Carbon Fiber/Carbon Nanofilament Reinforcement

**DOI:** 10.3390/ma7064182

**Published:** 2014-05-28

**Authors:** Mehran Tehrani, Ayoub Yari Boroujeni, Claudia Luhrs, Jonathan Phillips, Marwan S. Al-Haik

**Affiliations:** 1 Department of Mechanical Engineering, University of New Mexico, Albuquerque, NM 87131, USA; E-Mail: mtehrani@unm.edu; 2 Department of Engineering Science and Mechanics, Virginia Tech, Blacksburg, VA 24061, USA; E-Mail: yari@vt.edu; 3 Mechanical and Aerospace Engineering Department, Naval Postgraduate School, 700 Dyer Rd, Monterey, CA 93943, USA; E-Mail: ccluhrs@nps.edu; 4 Physics Department, Naval Postgraduate School, 833 Dyer Rd Monterey, CA 93943, USA; E-Mail: jphillip@nps.edu

**Keywords:** carbon fiber, carbon nanofilaments, fiber reinforced composites, mechanical properties

## Abstract

Carbon nanofilament and nanotubes (CNTs) have shown promise for enhancing the mechanical properties of fiber-reinforced composites (FRPs) and imparting multi-functionalities to them. While direct mixing of carbon nanofilaments with the polymer matrix in FRPs has several drawbacks, a high volume of uniform nanofilaments can be directly grown on fiber surfaces prior to composite fabrication. This study demonstrates the ability to create carbon nanofilaments on the surface of carbon fibers employing a synthesis method, graphitic structures by design (GSD), in which carbon structures are grown from fuel mixtures using nickel particles as the catalyst. The synthesis technique is proven feasible to grow nanofilament structures—from ethylene mixtures at 550 °C—on commercial polyacrylonitrile (PAN)-based carbon fibers. Raman spectroscopy and electron microscopy were employed to characterize the surface-grown carbon species. For comparison purposes, a catalytic chemical vapor deposition (CCVD) technique was also utilized to grow multiwall CNTs (MWCNTs) on carbon fiber yarns. The mechanical characterization showed that composites using the GSD-grown carbon nanofilaments outperform those using the CCVD-grown CNTs in terms of stiffness and tensile strength. The results suggest that further optimization of the GSD growth time, patterning and thermal shield coating of the carbon fibers is required to fully materialize the potential benefits of the GSD technique.

## 1. Introduction

Fiber-reinforced polymer plastics (FRPs) possess superior specific strengths and stiffness in comparison to other structural composites, such as metal or ceramic-reinforced composites. The relative ease of manufacturing, light weight, wide range of physical properties and high corrosion resistance make FRPs very desirable for several applications [1,2].

Compared to other structural fibers, carbon fibers are utilized when fatigue resistance, moderate strength and electrical conductivity are needed and when weight savings are crucial. Recently, nanofilament forms of carbon reinforcement, such as carbon nanotubes (CNTs) and carbon nanofibers [3], have gained growing interest in the composites community, due to their attractive mechanical properties. However, researchers have attempted to incorporate CNTs in polymer matrices and have met limited success, due to the extreme difficulty in uniformly dispersing CNTs in polymeric matrices, because of the large surface area of CNTs [4]. The high-aspect ratio CNTs tend to entangle and form agglomerates when dispersed into a viscous polymeric matrix. Sonication [3] and calendaring [5] have been employed extensively to mitigate this problem, but both techniques are not feasible beyond ~3.0 wt% CNT, due to the formation of aggregates [6]. A combination of dispersion and extrusion techniques have been reported in the literature for producing CNT composites [6] with a tailored microstructure, e.g., aligned CNTs. However, in both dispersion and extrusion techniques, producing uniform and well-dispersed CNT composites is difficult, due to the phase separation stemming from the stronger van der Waals interactions amongst the CNTs bundles compared with that between the CNTs and the polymer matrix [7]. Furthermore, excessive sonication of CNTs toward better dispersion might result in breaking them into shorter tubes, thus reducing their aspect ratios [8] and, consequently, negatively affecting their derived composite mechanical performance.

One viable alternative to prevent nanofilaments agglomeration is to anchor one end of the nanofilament to the substrate, thereby creating a stable multiscale structure. This approach can be implemented by physically growing the nanofilaments directly on the surface of the substrate (in this study, the substrate is micro-scale carbon fiber bundles). Carbon nanotubes have been grown on most substrates, such as silicon, silica and alumina [9]. However, there are fewer reports discussing CNT growth on carbon materials; in particular, yarns and fabrics [10]. Two challenges face CNT growth on carbon substrates, namely: (i) the transition metals that catalyze the CNTs growth can easily diffuse into the carbon substrates and; (ii) different phases of carbon materials are able to form on the graphite substrates, because the CNT growth conditions are identical to the graphite or diamond-like carbon growth [11].

Recently, carbon nanofilaments were grown on carbon fibers, both polyacrylonitrile- (PAN) and pitch-based, by hot filament chemical vapor deposition (HFCVD) using H_2_ and CH_4_ as precursors [12]. Nickel clusters were electrodeposited on the fiber surfaces to catalyze the growth, and uniform CNT coatings were obtained on both the PAN and pitch-based carbon fibers. Multi-walled CNTs with smooth walls and low impurity content were also grown on a carbon fiber cloth using plasma-enhanced chemical vapor deposition (PECVD) from a mixture of acetylene and ammonia [13]. In this case, a cobalt colloid was utilized to achieve the good coverage of nanofibers on the carbon cloth. The main draw back to CNT growth via CVD is the damage induced on the carbon fiber surface due to the high-temperature synthesis (>750 °C) [11,12]. Qu* et al.* [14] reported a new method for the uniform deposition of CNTs on carbon fibers. However, this method requires processing at 1100 °C in the presence of oxygen, and such a high temperature is anticipated to severely damage the carbon fibers. One approach to circumvent the thermal damage due to the synthesis at elevated temperature environments is to utilize ceramic-based thermal barrier coatings, such as SiC or SiO_2_.

In this study, carbon nano-filaments were grown utilizing a moderate temperature (*i.e.*, 550 °C) under atmospheric pressure. This atmospheric pressure process, derived from the process, graphitic structures by design (GSD) [15,16], is rapid, and the temperature is low enough (*i.e.*, 550 °C) to avoid severe structural damage to the substrate macroscale carbon fibers; and, the process is inexpensive and readily scalable. The GSD process does not utilize any toxic hydrocarbons or catalysts (e.g., xylene and metallocene) unlike CCVD [17]. Finally, GSD could offer the opportunity to place CNTs in pre-designated locations (where the catalyst is pre-deposited), whereas utilizing the CCVD technique, CNTs grow everywhere. This investigation sheds some light on the effect of the growth technique on the quasistatic mechanical properties of FRPs made out of a hybrid reinforcement that utilizes carbon nanofilaments grown on the surface of carbon fibers. 

## 2. Results and Discussion

**Figure 1:**
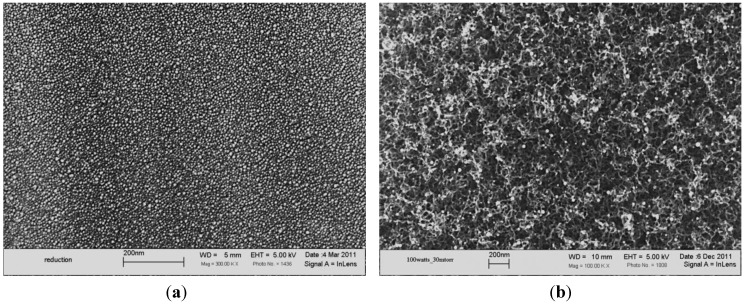
SEM micrographs for (**a**) nickel particles formed after the reduction process under N_2_-H_2_ at 550 °C; (**b**) nanofilaments grown utilizing the graphitic structures by design (GSD) technique with nickel particles attached to their tips.

Based on previous experiences, the nickel film should be fragmented into particles to grow carbon nanofilaments via GSD; otherwise, it might lead to the growth of either graphene of graphite [18]. A reduction step at 550 °C under a N_2_-H_2_ environment was carried out for 2 h under atmospheric pressure to fragment the nickel film into nanometer-sized particles and to remove any nickel oxides (Figure 1a). These particles are retained on the tips of the nanofilaments grown via GSD, as shown in Figure 1b.

**Figure 2:**
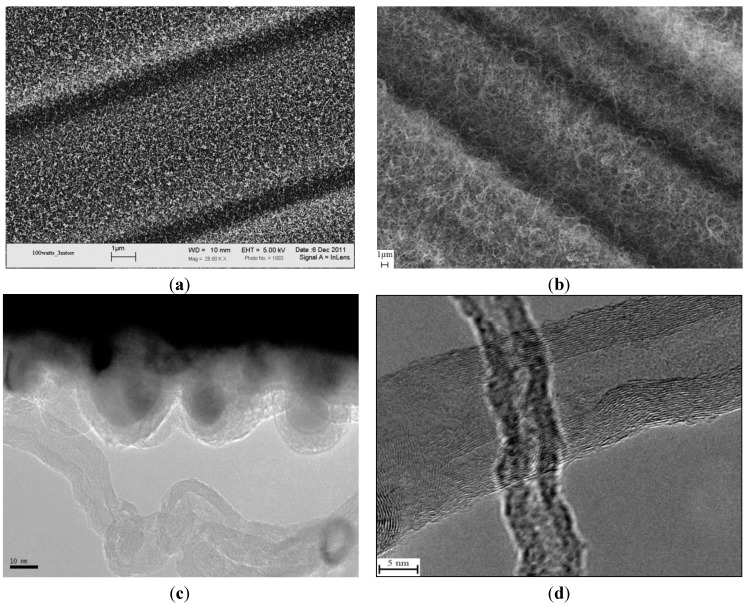
The SEM micrograph of nanofilaments grown via (**a**) GSD and (**b**) chemical vapor deposition (CCVD); The TEM micrograph of the morphologies of nanofilaments grown via (**c**) GSD and (**d**) CCVD.

The SEM micrographs in Figure 2a,b exhibit a uniform growth of CNTs utilizing GSD and CCVD techniques, respectively, on the surface of PAN-based carbon fibers, where the nickel catalytic particles were deposited (for the case of GSD). Both fibers were precoated with 75 nm-thick films of SiO_2_, in anticipation of better thermal shielding against the synthesis temperature. The morphologies of the grown nanofilaments are shown in the TEM micrographs (Figure 2c, d). The GSD-synthesized nanofilament does not exhibit well-defined walls and possesses a diameter of less than 20 nm, whereas the CCVD yielded better defined multiwall CNTs (MWCNTs) with variable diameters.

**Figure 3:**
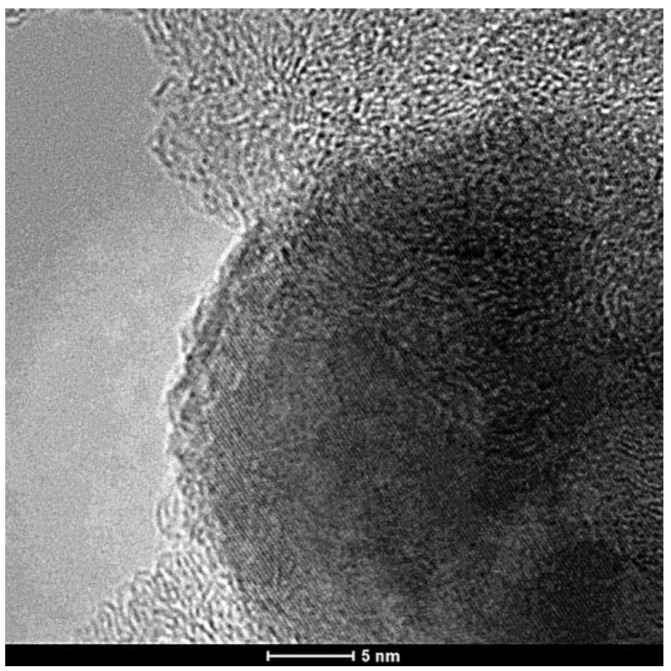
The TEM micrograph of a nickel particle from which a carbon nanofilament was grown.

Since the samples comprising the surface-grown nanofilaments were exposed to elevated temperatures (*i.e.*, 550–680 °C), reference samples of raw fabric and SiO_2_ sputter-coated fabric were exposed to an identical thermal environment similar to GSD (except for prohibiting the growth without the flowing of hydrocarbon gas) for later comparison. These samples are referred to as “heat treated” throughout this study. The Raman spectra were measured for the raw PAN-based carbon fabric, SiO_2_ sputter-coated fabric, heat-treated fabric, heat-treated SiO_2_ sputter-coated fabric and samples with CNTs grown on their surfaces via the GSD and CCVD techniques. While the as-sputtered nickel thin film is amorphous, evidenced by the ordered fringe patterns of Figure 3, upon fragmentation and reduction, the nickel particles become crystalline.

For carbonaceous materials, the Raman spectra exhibit two distinct bands. The disorder induced D band at 1354.7 cm^−1^ in the MWCNT spectra and between 1330 and 1390 cm^−1^ in single wall carbon nanotubes (SWCNT) The tangential mode G band, related to the ordered graphitic structure, appears at 1581.2 cm^−1^ for the MWCNT and between 1595 and 1605 cm^−1^ for the SWCNT [19]. 

**Figure 4:**
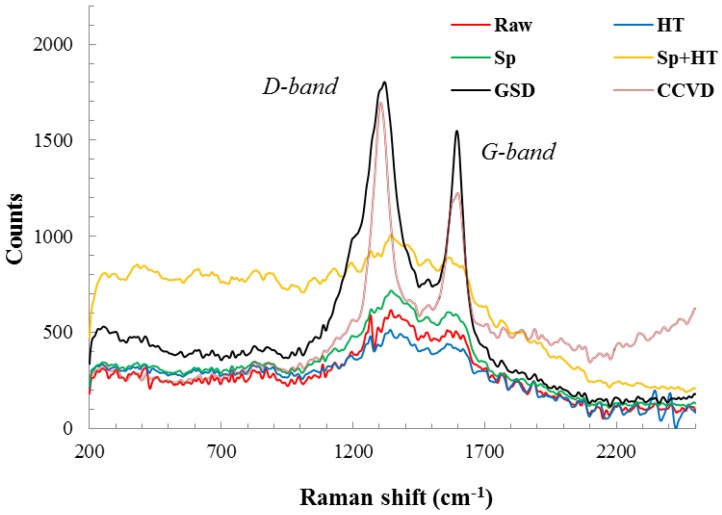
Raman spectra of the surfaces of different processed carbon fibers. Specimens are based on raw polyacrylonitrile (PAN)-based carbon fabric (Raw), SiO_2_ sputter-coated fabric (Sp), heat-treated fabric (HT), SiO_2_ sputter-coated then heat-treated fabric (Sp + HT), and with CNTs grown on their surfaces via graphitic structures by design (GSD) and catalytic chemical vapor deposition (CCVD).

Raman peaks (Figure 4) for all of the samples without nanofilament growth are very weak, regardless of the prior surface treatment, and do not exhibit the presence of a crystalline form of carbon. The raw carbon fibers did not display significant peaks. It is well known that PAN-based carbon fibers do not exhibit the G band (unlike graphitic pitch-based fibers); rather, they exhibit the turbostratic appearance of the D line, which corresponds to the structural disorder caused by the existence of the sp^3^ bonds [20].

Sputtering the fibers with nickel and SiO_2_ films assisted in contrasting the G band for the fibers. Furthermore, heat treating of the samples with the deposited films made the D and G bands more pronounced. The CNTs grown on the surfaces of the PAN-based carbon fiber fabric via GSD or CCVD demonstrate the D-band center value at 1350 cm^−1^ and the G-band at 1580 cm^−1^, respectively; in good agreement with those of the Raman spectra of MWCNTs. Although these peaks are also observed for graphite [21], it is evident from the SEM and TEM micrographs of the nanofilaments that the Raman peaks cannot be from graphite.

The intensity ratio of the two bands (I_D_/I_G_) can be conceded as a quantitative measure of the amount of structurally-ordered graphite crystallite in the carbonaceous material. From Figure 4, the decrease in the intensity ratio (I_D_/I_G_) was more noticeable for the carbon fibers with MWCNTs grown by CCVD. This reveals that the degree of crystallinity of the MWCNTs grown using CCVD is higher than that for the nanofilaments grown via GSD. Moreover, the width of the D peak for the CCVD sample is narrower than that for the sample prepared via GSD. This is indicative of a higher degree of order in the MWCNTs prepared via CCVD compared to the nanofilaments grown via GSD, which was confirmed by the TEM images, Figure 2c,d.

The stiffness of the two-lamina composites is governed by the carbon fiber’s core and, hence, is less likely to be affected by the elevated temperatures of CCVD and GSD if an inert atmosphere is employed. However, the tensile strength is highly influenced by the quality of the surface of the carbon fibers and the strength of the adhesion (uniform stress transfer) between the matrix and the fibers. Therefore, the ultimate tensile strength is expected to be affected by the surface coating, heat treatment and the growth time.

**Figure 5:**
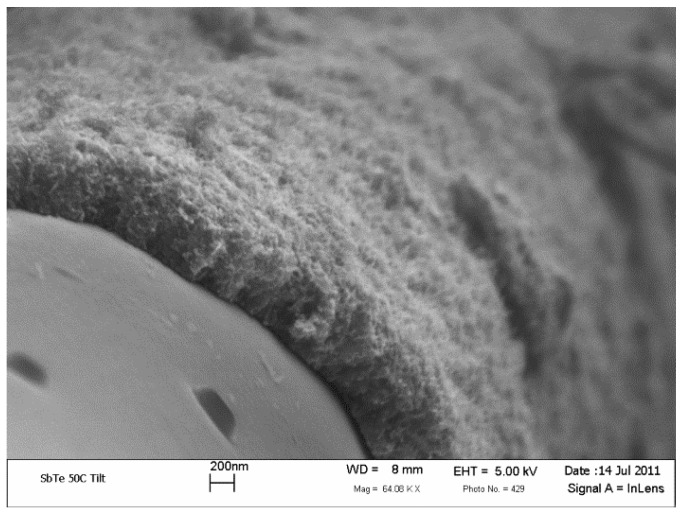
Cross-sectional SEM micrograph of a carbon fiber grafted with surface-grown carbon nanofilament utilizing the GSD technique.

The morphology, length and density of the grown CNTs or nanofilaments are postulated to affect the polymer matrix infusion into the CNT or nanofilament layer and, subsequently, the interfacial bonding at the fiber/epoxy interface. Figure 5 shows SEM micrograph of a cross-sectional view of carbon fiber covered with a dense layer of GSD-grown nanofilament. The thickness of the layer (1 h growth time) is almost 0.5 microns. The length of the grown CNTs can be controlled by the initial thickness of the nickel layer and the growth time. The filament layer seems to be coherent and connected to the surface of the PAN carbon fibers. However, to attain a good adhesion between the polymer/fiber, it is imperative that the polymer matrix infuses into the dense CNT forest. 

**Figure 6:**
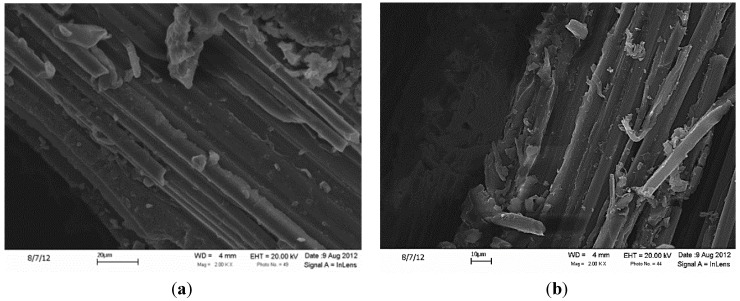
Polymer matrix penetration of the region adjacent to the nanofilaments for the composites based on the (**a**) CCVD and (**b**) GSD methods.

**Figure 7:**
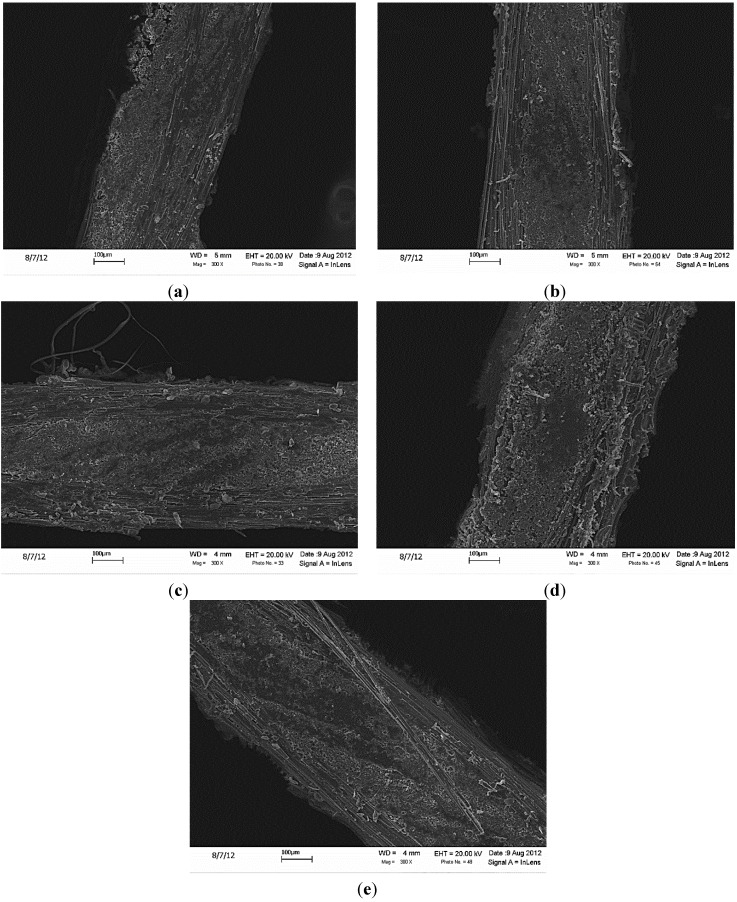
The cross-sections of all fabricated composite samples were investigated under scanning electron microscopy (SEM). The cross-sectional images of all samples, as well as the magnified images for the GSD and CCVD samples are shown below. The SEM micrographs show the same level of matrix penetration for all samples. (**a**) R; (**b**) R + HT; (**c**) Sp + HT; (**d**) GSD; (**e**) CCVD.

The carbon monofilaments only grew on the exposed fiber bundles on the upper and lower surfaces of the woven carbon fabrics (*i.e.*, what will be the ply interfaces in a composite lamina). However, they did not influence the polymer matrix penetration to the regions adjacent to the grown nanofilaments (see Figure 6). As observed from the SEM micrographs of the cross-sections of different samples (Figure 7), the overall penetration of the matrix seems to be identical for all the samples. However, while grafting of CNTs was achieved with a minimal weight penalty, it affected the volume fractions of the composite panels. The volume fractions for different composite configurations are summarized in Table 1.

The tensile test results were, therefore, normalized with respect to the corresponding volume fractions according to the composites rule of mixture. Although the rule of mixture does not provide the most accurate prediction of tensile properties, it is employed here to justify the comparison between the different samples and to provide a better contrast of the effects of the different surface treatments on the tensile properties. The results of the tension tests are summarized in Table 2.

**Table 1:** Fiber volume fractions for the specimens based on raw PAN-based carbon fabric (Raw), SiO_2_ sputter-coated fabric (Sp), heat-treated fabric (HT), SiO_2_ sputter-coated then heat-treated fabric (Sp + HT) and with CNTs grown on their surfaces via graphitic structures by design (GSD) and catalytic chemical vapor deposition (CCVD).

Composite Sample	Label	*V*_f_ (%)
Raw	R	56
Heat treated	HT	57
SiO_2_ sputter-coated	Sp	55
SiO_2_ sputter-coated then heat treated	Sp+HT	54
CNT grown with GSD	GSD	49
CNT grown with CCVD	CCVD	49

**Table 2:** Tensile mechanical properties for the specimens based on raw PAN-based carbon fabric (Raw), SiO_2_ sputter-coated fabric (Sp), heat-treated fabric (HT), SiO_2_ sputter-coated then heat-treated fabric (Sp + HT) and with CNTs grown on their surfaces via GSD and CCVD.

Fiber’s configuration	Young’s modulus (GPa)	Tensile strength (MPa)
Raw	40.4 ± 1.2	606 ± 31
HT	41.4 ± 0.9	556 ± 44
Sp	40.6 ± 3.6	547 ± 42
Sp + HT	44.1 ± 1.4	557 ± 27
GSD	43.7 ± 2.6	585 ± 37
CCVD	38.9 ± 4.8	184 ± 10

The normalized mechanical properties (Table 2) indicate that the stiffness of the composite was retained upon exposure to moderate temperatures (*i.e.*, 550 °C in N_2_ atmosphere). This observation is manifested by the heat-treated fabric (HT) and the HT and SiO_2_ sputter-coated fabric (HT + Sp) samples. The surface alteration via coating with SiO_2_ and the heat treatment or the presence of GSD-CNTs on the surface resulted in an increase in the stiffness (see Table 2). However, the sample with CCVD-grown CNTs on the SiO_2_ layer exhibited a slight degradation of almost 4% in the stiffness of the corresponding composite. This degradation can be attributed to the higher temperature of the CCVD reaction that accelerates carbon diffusions and partially deteriorates the carbon fiber and, to a lesser extent, to the poor adhesion between the epoxy/fibers and CNT/fiber. 

Heat treatment of the carbon fabric also leads to the removal of the sizing and, thus, alteration of the surface of the fibers. Removing the sizing through heat treatment resulted in an 8% reduction in the strength of the composite compared to the reference composite with the sizing intact (Table 2). The introduction of the SiO_2_ layer (Sp sample) lessens the bonding between the epoxy and fibers and, thus, induces a slight reduction in the strength of the composite. The heat treatment of the sputter-coated fabrics (see the Sp + HT sample) does not appear to influence the adhesion of fiber/matrix and the strength of the composites; the strength of the Sp and the Sp + HT composites were virtually identical. Grafting the nanofilaments directly onto the fibers allows for the placement of high volume fractions of un-agglomerated nanofillers. This volume fraction is far larger than what can be effectively achieved when CNTs are pre-mixed with the epoxy matrix (typically within 3% for proper dispersion). It is speculated that the highly viscous matrix (viscosity of 950 cps) might not be efficient in fully impregnating the dense surface-grown nanofilament forests. Conversely, the enhanced fiber/matrix adhesion due to the interactions between the surface grown CNTs and the polymer matrix yielded an improved stress transfer to the fibers compared to all other samples with modified surfaces (*i.e.*, Sp, Sp + HT and HT). The composite based on the GSD grown nanofilaments counterbalanced some of the undesired effects from the presence of the SiO_2_ layer and heat treatment on the strength. While the strength of the GSD samples improved by 5% compared to the Sp + Ht ones, it was still 3.5% lower than the strength of the reference samples. Nevertheless, the harsh thermal environment of CCVD (*i.e.*, exposure to the temperature of 680 °C) causes drastic degradation of the fibers due to temperature-accelerated diffusion and oxidation of carbon, which deteriorates the carbon fiber core and surface. Moreover, the surface of the fibers is malformed due to the presence of nanofilaments (possibly not fully attached to the fiber) on the surface of the SiO_2_-coated carbon fibers. Test coupons based on FRPs with CCVD surface-grown CNTs ruptured in a very brittle manner. A 70% decrease in the strength of the composites based on CCVD-CNTs can be primarily attributed to the severe degradation of the fiber surfaces, due to the elevated synthesis temperatures. Among the tested samples, only the SiO_2_-coated (Sp) and SiO_2_-coated/heat-treated (Sp + HT) samples exhibited delamination during the tension tests.

The carbon fiber samples were weighed pre- and post-processing in the synthesis reactor. The measurements were repeated for the samples with SiO_2_ coating only (the Sp + HT sample was considered as the reference for calculating the weight of CNTs) and for the samples with SiO_2_ coating and nickel catalyst (the ones that CNTs grow on later). Utilizing a digital scale with an accuracy of ±0.0001 g, we conclude that for both samples processed via GSD and CCVD, the weight fractions of the CNTs in the composite is less than 0.05 wt%.

The hand layup process that was used for the fabrication of the panels does not effectively control the volume fractions of composites. However, the normalized tensile test results confirmed the hypothesis that the core of the fibers is unaffected by the elevated reaction temperatures of both GSD and CCVD. Among all the samples, the tensile strength of the panels fabricated from the CCVD-processed fabrics degraded significantly. Thermally-induced surface damage to the fiber surface and the weak interaction between the surface CNTs and polymer matrix are the main reasons for the mechanical weakening of the CCVD composite samples. The conclusion that can be drawn is that the CCVD technique allows for the placement of high-quality crystalline CNTs (compared to the nanofilaments grown via GSD) at the price of significantly degrading the mechanical properties, due to the severe substrate fiber damage.

The reduction of tensile properties of the GSD samples (Table 2) is at worst 3.5% for the strength. The CCVD yielded even more significant reductions of 4% and 70% for the modulus and the strength, respectively. The degradation of the composites properties due to thermal annealing of the base carbon fibers during CCVD was observed by several other groups. For example, when utilizing CCVD for growing CNTs on the surface of IM7 carbon fibers (much stronger and denser than the AS4 fibers) at 750 °C for 1 h, Qian* et al.* [22] reported a 15%–25% reduction on the tensile strength. Zhang* et al.* [17] performed a CCVD to grow CNTs over T650 and IM7 carbon fibers and observed that the strength of the corresponding composite based on T650 fibers with grafted CNTs compared to composite based on the raw fibers drops by 46% when the growth temperatures was 800 °C. In contrast, under the same growth conditions, the composites based on IM7 carbon fibers with grafted CNTs exhibited a reduction of 70% of the original strength.

## 3. Experimental Section 

### 3.1. Growing Carbon Nanofilament Using GSD

Commercial PAN-based plain-woven carbon fabric (AS4 supplied by HEXCEL Inc., Stamford, CT, USA) was utilized as the substrate to grow carbon nanofilaments. Samples of 13 × 13 cm^2^ were cut from the raw fabric, some of which were reserved intact for the next step as reference samples. In anticipation of shielding the carbon yarn against the elevated temperatures encountered during the growth procedures and to prevent undesired carbon diffusion, thin films of thermal coating (75 nm thick SiO_2_) were sputtered on the fabric top and bottom surfaces [23]. The catalyst in the form of a 2 nm-thick film of nickel was sputtered on top of the SiO_2_ layers. A magnetron sputtering system (ATC Orion high vacuum sputtering system, AJA International Inc., Scituate, MA, USA) was employed to sputter both the SiO_2_ and nickel films on the PAN-based carbon fiber fabrics. The sputtering process was carried out with an argon gas flow at 300 Watts power from a radio frequency (RF) source (to deposit the 75-nm SiO_2_ film on the surface of the carbon fibers) and a DC source (to deposit the 2-nm nickel film on top of the SiO_2_ film,) both at 3 millitorr vacuum.

In order to grow carbon nanofilaments on the fabrics coated with SiO_2_ and nickel films, a tube furnace reactor was utilized. The furnace was comprised of a 7.62-cm diameter quartz tube and a 45.72-cm heating zone. The first step of the growth process involves flushing the tube with nitrogen and vacuuming it with a mechanical pump while the nitrogen was flowing. This step ensures the elimination of oxygen inside the reaction tube.

The CNT growth was initiated as a mixture of N_2_/H_2_/C_2_H_4_ and was introduced while maintaining the temperature at 550 °C. The growth time was set to one hour. The hydrocarbon (ethylene) undergoes a homogeneous reaction over the nickel catalyst in the presence of H_2_, and the carbon radicals get deposited in the form of nanotubes [18,24,25]. The furnace was cooled down to ambient temperature under an inert nitrogen environment.

To provide better insight into the effect of the nanofilament growth condition and synthesis method, MWCNTs were also synthesized on separate yarns by catalytic chemical vapor deposition (CCVD) in a simple hot-wall reactor at ambient pressure. In this process, the catalyst, ferrocene, was dissolved in a liquid hydrocarbon, xylene, to form a feed solution. This solution was delivered by a syringe pump to an injection tube and dispersed into a flow stream of hydrogen and helium. This vapor was transported to a hot quartz tube reactor. Carbon nanotubes were grown on SiO_2_-coated woven carbon fabric at 680 °C for 1 h.

### 3.2. Microstructural and Mechanical Characterization

A 5200 Hitachi SEM (Tokyo, Japan) and a Titan 300 TEM (FEI, Inc., Hillsboro, OR, USA) operated at 5 and 300 keV, respectively, were utilized to examine the synthesized MWCNTs. Raman spectra were obtained utilizing a ProSeek Raman system from Raman System, Inc. (Woburn, MA, USA) These spectra were obtained with a confocal Raman microscope, using a 5-mW, 785-nm excitation wavelength laser beam focused on the sample with a 50× objective. Spectra were obtained as the sum of 30-s integration time.

For the purpose of mechanical testing, a set of flat, two-layer composite lamina of 12.5 × 12.5 cm^2^ were manufactured using vacuum and a press-assisted hand lay-up process. The lay-up stack comprised a vacuum bag, peel ply release fabric, stacked carbon fabrics impregnated with the epoxy, another peel ply film, perforated release film and breather cloth, sequentially. The lay-up set was vacuum bagged, while a pressure of 5 kN via a dead weight was applied to it. Simultaneous use of the vacuum and dead weight assured the degassing and curing of the resin, while the carbon fabric stack was kept intact under high pressure. The composite was left to cure under room temperature for 24 h. The matrix material was Aeropoxy™ manufactured by PTM&W Industries, Inc. (Santa Fe Springs, CA, USA) This epoxy system was used to manufacture both FRPs [26,27] and composites based on SWCNT [7] and MWCNT [28]. Abraded G-10 tabs were attached utilizing the Aeropoxy to the ends of the tensile specimen. Tensile test coupons of 12.5 × 1.25 cm^2^ were cut using a table saw. The ultimate tensile strength and Young’s modulus for the specimens were measured from tension tests utilizing an Instron^®^ 4400R frame (Instron, Inc. Norwood, MA, USA). The tensile tests were performed according to the ASTM standard D3039/D3039M-08 [29] under a constant cross-head speed of 2 mm/min until failure occurred.

The strain was measured using an extensometer (MTS Testing Systems, Inc., Eden Prairie, MN, USA). A total of eight samples for each configuration were tested to determine the tensile properties.

## 4. Conclusions 

The surface modification of the carbon fiber through heat treatment, coating with SiO_2_ film in an attempt to prevent the fibers’ thermal degradation and growing carbon nanofilaments via GSD and CCVD on the surface influence the mechanical properties of the composites based on these different fiber configurations. The mechanical testing showed that composites using the GSD-grown nanofilaments outperform those using the CCVD-grown CNTs in terms of stiffness and tensile strength. The conclusion that can be drawn is that the CCVD technique allows for the placement of higher quality CNTs (compared to GSD) at the compromise of significantly degraded mechanical properties. The tensile results indicated that the SiO_2_ thin film protected the PAN-based carbon fiber against undesired diffusions and the temperatures utilized in GSD technique (550 °C), but were not capable of shielding the fibers at higher temperatures used in the CCVD method. The reduction in the strength encountered by the fibers via the GSD growth is not desirable and, yet, are minimal (only a 3.5% reduction in strength compared to the reference samples). This trend was observed by the different groups who have utilized CCVD to grow CNTs on the surface of carbon fibers. Therefore, it is believed that the GSD still needs further optimization in terms of the growth temperature and the shielding of the base fibers.
